# Increased incidence of thyroid disease in patients with sarcoidosis: a systematic review and meta-analysis

**DOI:** 10.1530/EC-23-0157

**Published:** 2023-08-01

**Authors:** Caihong Xin, Lijuan Niu, Huaying Fan, Jing Xie, Xin Sun

**Affiliations:** 1 Department of Endocrinology and Metabolism, The Fourth People’s Hospital of Shenyang, Shenyang, P.R. China; 2 Department of Endocrinology and Metabolism, The First Affiliated Hospital of Soochow University, Suzhou, P.R. China

**Keywords:** thyroid disease, sarcoidosis, incidence

## Abstract

**Background:**

Sarcoidosis is a multiple systemic granulomatous disease, and its main pathological feature is non-caseous necrotic epithelial granuloma. The pathogenesis is not fully understood. The prevalence of thyroid disease is likely higher among individuals with sarcoidosis. However, this association still lacks clinical evidence.

**Objective:**

The aim of this study was to estimate the incidence of thyroid disease in patients with sarcoidosis.

**Methods:**

A literature search was conducted using PubMed, Web of Science, Embase, and China National Knowledge Infrastructure literature databases. Fixed- or random-effects models were used for analysis according to heterogeneity. The results were subjected to meta-analysis with odds ratios (ORs) and corresponding 95% confidence intervals (CIs).

**Results:**

In total, six articles were included in this meta-analysis, which involved 2044 sarcoidosis cases and 5652 controls. The studies found that the incidence of thyroid disease in patients with sarcoidosis was significantly increased compared to the controls (OR 3.28, 95% CI 1.83–5.88).

**Conclusions:**

This systematic review is the first to evaluate the incidence of thyroid disease in sarcoidosis patients, which was increased compared with the controls, suggesting that sarcoidosis patients should be screened for thyroid disease.

## Introduction

Sarcoidosis is a multiple systemic granulomatous disease (1). Its main pathological feature is non-caseous necrotic epithelial granuloma, which affects the lung, lymph nodes, and other organs. At present, the pathogenesis of sarcoidosis is unclear, and the existing research supports that individuals with genetic susceptibility have granulomatous reactions due to immune dysfunction caused by special antigens (2, 3). The clinical manifestations of sarcoidosis are complex and diverse, without specific. The common symptoms of sarcoidosis patients are bilateral hilar lymphadenopathy, with (or without) diffuse changes in the pulmonary interstitium. Other symptoms are often manifested in the skin, eyes, heart, liver, spleen, and lymph nodes. Some patients are diagnosed in the physical examination without obvious clinical symptoms (4).

The first case of sarcoidosis and thyroid disease was reported in 1938 (5). Recently, studies about the relationship between sarcoidosis and thyroid disease have increased. Several studies have revealed a high prevalence of thyroid disease in sarcoidosis patients (6, 7, 8, 9, 10, 11, 12). However, most of these studies were cross-sectional surveys and lacked agreement. This meta-analysis aims to summarize the incidence of thyroid disease in patients with sarcoidosis.

## Methods

### Search

The PROSPERO registration number of our meta-analysis is CRD42020154711. The following search terms were used for the title or abstract: ‘sarcoidosis’ in combination with the terms ‘thyroid disease’, ‘thyroiditis’, ‘thyroid antibody’, ‘hypothyroidism’, or ‘hyperthyroidism’. The search period was set between 1980 and 2022. We simultaneously traced the references of the collected relevant literature, searched for studies that met the inclusion criteria, and eliminated duplicate studies. This meta-analysis was performed in accordance with the PRISMA guidelines, and we have provided the PRISMA list in Supplementary File 1 (see section on supplementary materials given at the end of this article).

### Inclusion criteria

Only studies which met the following criteria were included in this meta-analysis: (i) all cases were confirmed by a doctor, not by a record or based on self-reports; (ii) case–control design; and (iii) sufficient data of cases and controls that could allow us to calculate the odds ratio (OR) with 95% confidence interval (CI) and a *P-*value. Reviews, case reports, letters, and animal research were excluded.

The thyroid disease group was defined as a group of patients with any abnormal thyroid function test or elevated thyroid antibodies. The thyroid antibody-positive group was defined as a group of patients with elevated thyroid antibodies. The clinical thyroid group was defined as patients with hypothyroidism or hyperthyroidism.

### Data extraction and risk of bias

Two researchers independently and simultaneously screened the literature based on the inclusion and exclusion criteria by reading article titles, abstracts, and full texts. A third researcher was asked to intervene and finalize the decision in cases of disagreement. The extracted data included author, year, survey area, sample source, research object characteristics, and sample size.

The Newcastle–Ottawa Scale (NOS) is a risk of a bias assessment tool for observational studies recommended by the Cochrane Collaboration (13). The quality of the included studies was evaluated according to the NOS. The NOS includes three aspects: the selection method of the case and control groups, the comparability of the case and control groups, and the evaluation method of exposure. The NOS ranged from zero to nine stars, and the quality was based on star scores.

Bias was also assessed with the ROBINS-I (risk of bias in non-randomized studies-of interventions) tool for nonrandomized studies. The ROBINS-I tool views each study as an attempt to emulate a hypothetical pragmatic randomized trial and assesses seven domains through which bias might be introduced: bias due to confounding, bias in the selection of participants into the study, bias in classification of interventions, bias due to deviations from intended interventions, bias due to missing data, bias in the measurement of outcomes, and bias in the selection of the reported result. The judgments within each domain carry forward to an overall risk of bias of: ‘Low’, ‘Moderate’, ‘Serious’, ‘Critical’ or ‘No information’ (14).

### Statistical analysis

OR with corresponding 95% CIs were determined as the results of the meta-analysis. Meta-analysis was conducted using Stata 12.0 software (College Station, TX, USA). First, a heterogeneity test was performed using the *I*^2^. If *I*^2^ < 50%, a fixed-effects model is used; If *I*^2^ ≥ 50%, heterogeneity is significant, and a random-effects model is used. The stability of the meta-analysis results was evaluated by a sensitivity analysis. Low-quality literature was excluded, and the impact of a single study on the overall research results was excluded for each study. Publication bias was tested using Begg’s test. Differences were considered statistically significant at *P* < 0.05.

## Results

**Figure 1:**
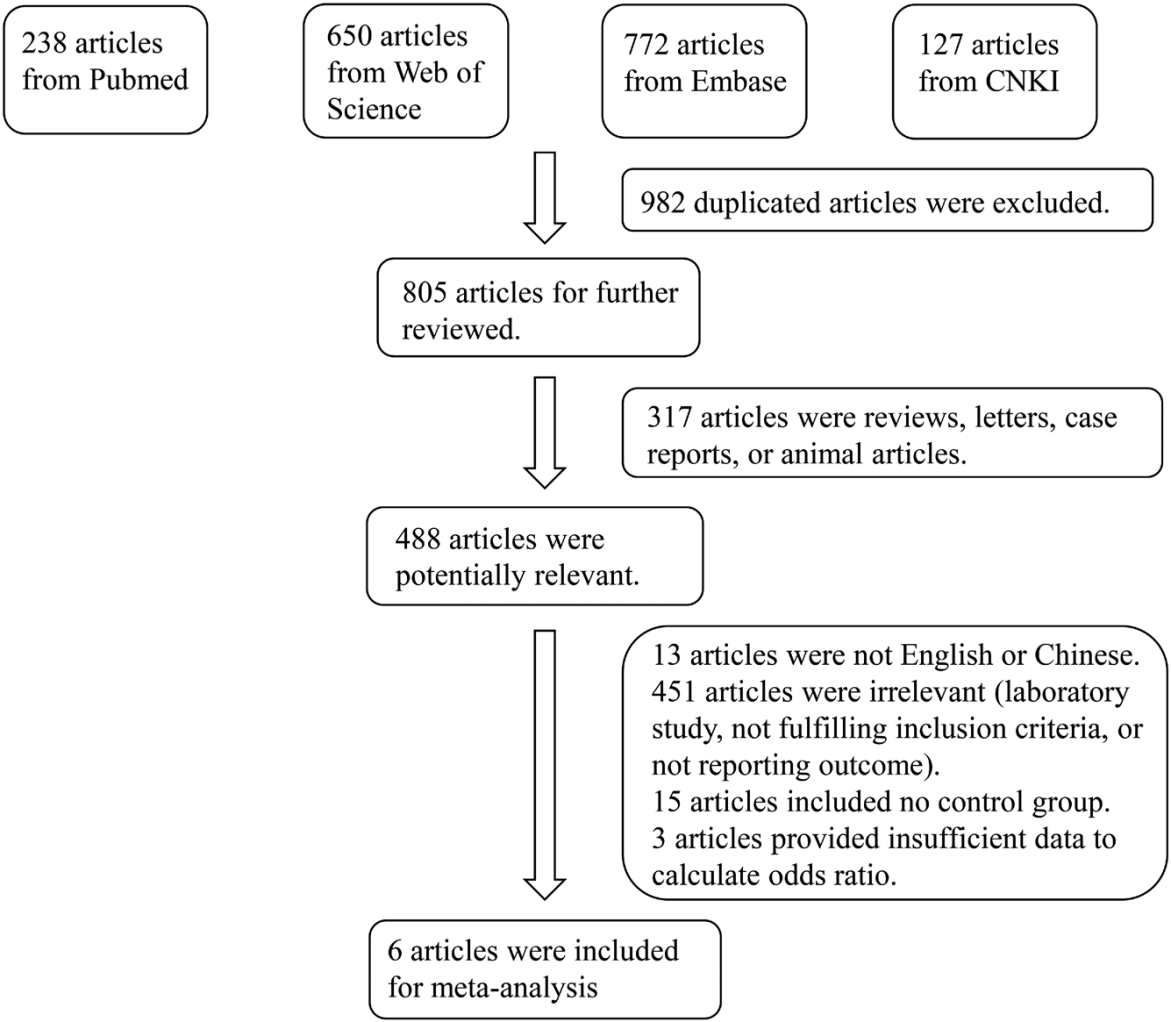
Flowchart showing the detailed procedure for the inclusion or exclusion of studies.

In total, 1787 studies were retrieved from PubMed, Web of Science, Embase, and CNKI databases. After screening, six articles were included in the meta-analysis (15, 16, 17, 18, 19, 20). The reasoning for inclusion during the full-text selection is shown in Fig. 1. Altogether, the six articles included 2044 sarcoidosis cases and 5651 controls. The characteristics of the selected studies are summarized in Tables 1 and 2. Overall, in accordance with the suggested criteria for Selection, Comparability, and Exposure categories of the NOS, the studies included in this meta-analysis were of acceptable quality. The ROBINS-I assessment of study bias for included studies was presented in Supplementary File 2.

**Table 1:** Study characteristics of the published studies included in the meta-analysis.

Study	Publication year	Study period	Region	Population	Case (*n*)	Control (*n*)	Case factor	Control factor	NOS
Nakamura *et al.* (15)	1997	-	Japan	Age ≥ 40 years old	45	170	34F 11M, 54.2 ± 6.9 years, the patients were diagnosed by a combination of clinical, radiographical, and histological findings	Control I: 88 employees (43F 45M, 49.3 ± 6.3 years) worked in the Hamamatsu University Hospital selected at random during a regular annual physical examination	7
								Control II: 82 company workers (17F 65M, 45.5 ± 5.5 years)	
Ilias *et al.* (16)	1998	-	Greece	Adults	26	26	19F 7M, 31–78 years, 57 ± 3 years, 21 patients were newly diagnosed, 5 patients presented with a reactivated disease	Age- and sex-matched patients with chronic obstructive pulmonary disease	7
Antonelli *et al.* (17)	2005	-	Italy	Adults	111	333	75F 36M, 47 ± 12 years, all patients were proven by biopsy	Age- and sex-matched subjects of the background population from the same geographic area	8
Malli *et al.* (18)	2012	2007–2010	Greece	Adults	68	75	45F 23M, 53.08 ± 11.6 years, all patients were proven by biopsy	59F 16M, 50.78 ± 13.57 years, age-matched healthy volunteers	8
Nowiński *et al.* (19)	2015	2007–2011	Poland	Adults	557	100	266F 291M, 48.43 ± 12.0 years, all patients were proven by biopsy	44F 56M, 49.25 ± 10.3 years, age-matched healthy volunteers	8
Wu *et al.* (20)	2016	1997–2010	China	Adults	1237	4948	776F 461M, 34–57 years, all patients diagnosed by dermatologists, ophthalmologists, or internal medicine specialists including rheumatologists and pulmonologists for at least three consecutive times in outpatient service	3104F 1844M, 34–57 years, age- and sex -matched healthy volunteers	8

NOS, Newcastle–Ottawa Scale.

**Table 2:** Details of thyroid disease in patients with sarcoidosis and controls in the study (n).

Study	Thyroid disease		Thyroid antibody positive				Clinical thyroid disease	
			TgAb positive		TPOAb positive			
	Case	Control	Case	Control	Case	Control	Case	Control
Nakamura *et al.* (15)	17	15	15	14	10	8	-	-
Llias *et al.* (16)	3	0	3	0	0	0	-	-
Antonelli *et al.* (17)	46	95	11	46	29	52	28	34
Malli *et al.* (18)	42	13	4	3	17	7	20	13
Nowiński *et al.* (19)	73	4	-	-	-	-	73	4
Wu *et al.* (20)	143	359	-	-	-	-	143	359

TgAb, thyroglobulin antibody; TPOAb, thyroid peroxidase antibody.

### Results of the meta-analysis

**Figure 2:**
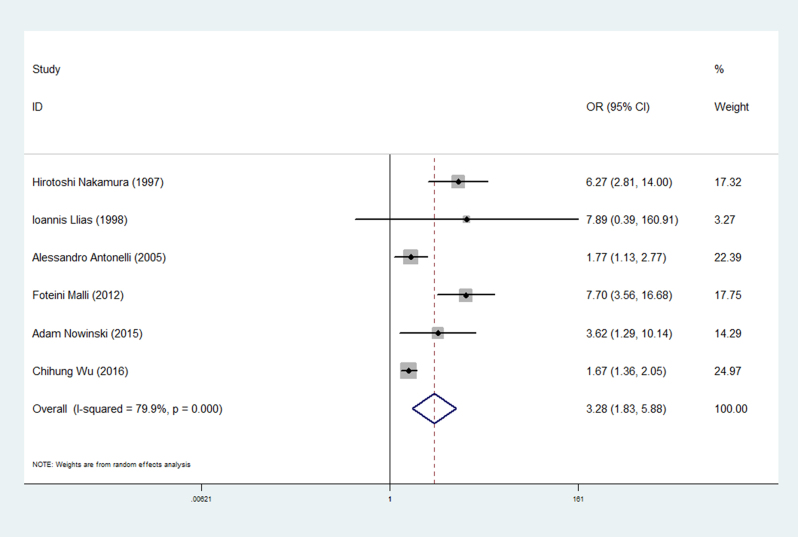
Forest plots of the frequency of thyroid disease in patients with sarcoidosis compared with the controls. The diamond represents the pooled OR and 95% CI.

**Figure 3:**
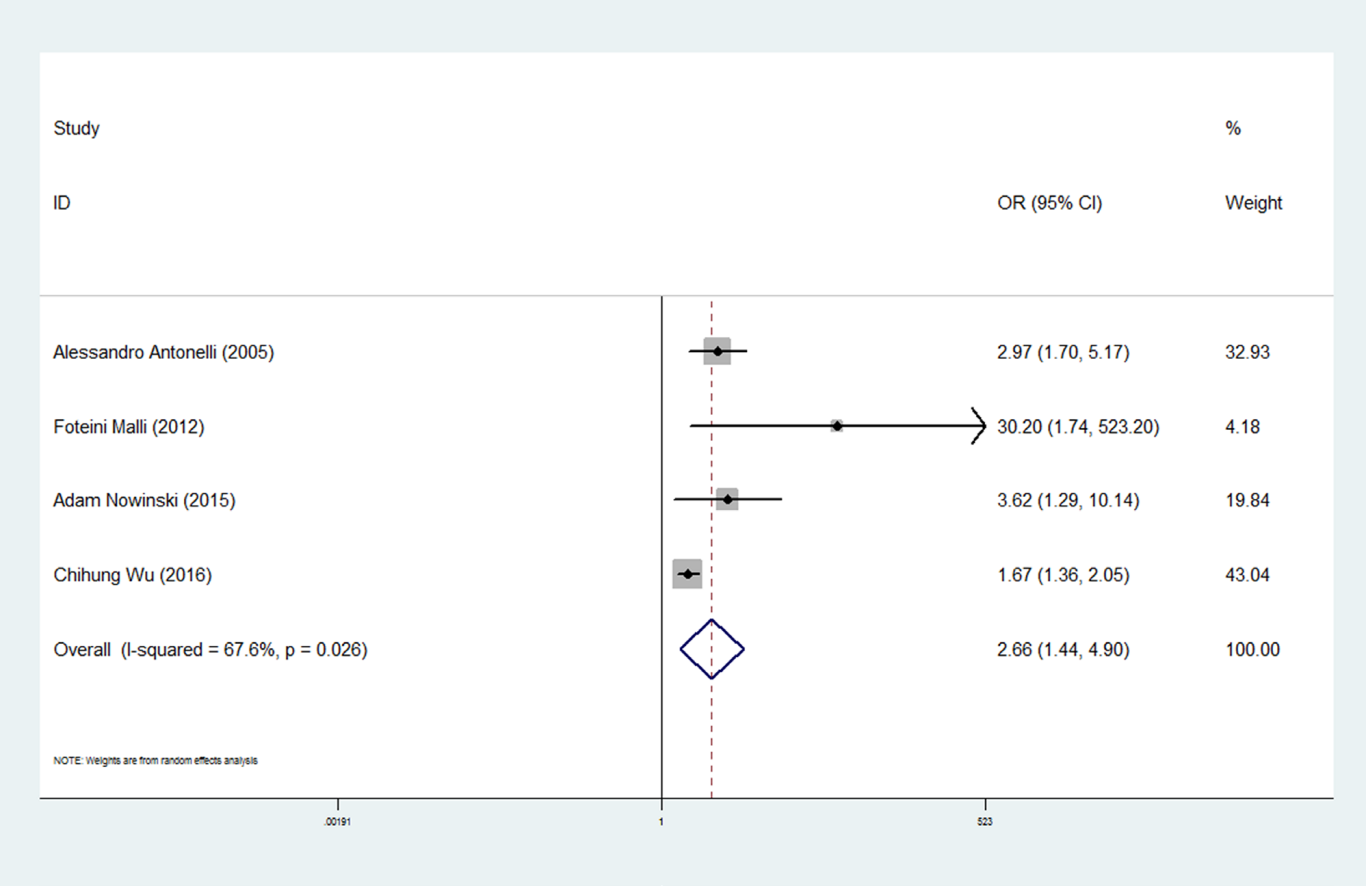
Forest plots of the frequency of clinical thyroid disease in patients with sarcoidosis compared with the controls. The diamond represents the pooled OR and 95% CI.

**Figure 4:**
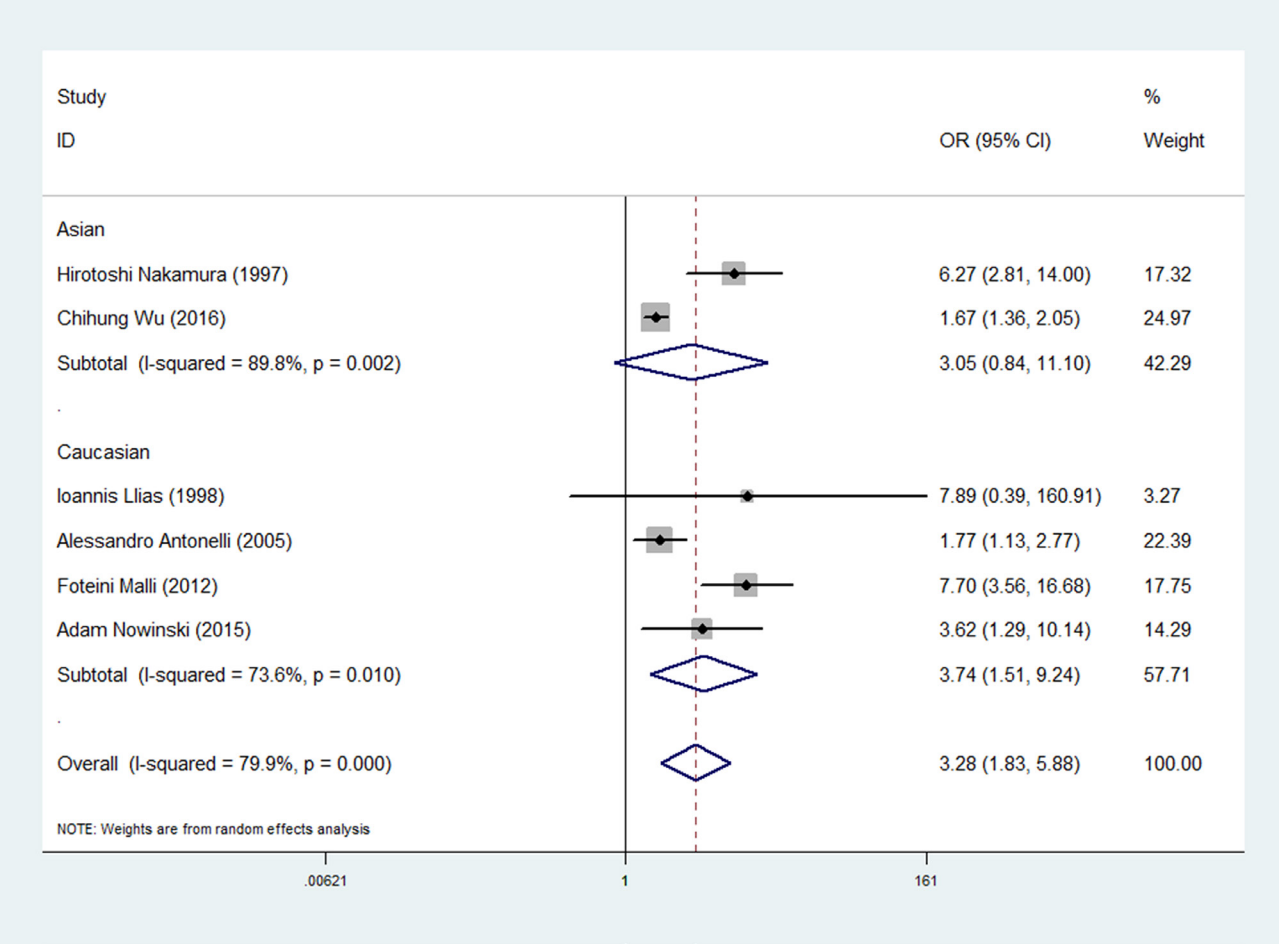
Forest plots of the different races in patients with sarcoidosis compared with the controls. The diamond represents the pooled OR and 95% CI.

The results of the meta-analysis showed that the prevalence of thyroid disease in patients with sarcoidosis was higher than that in controls (OR 3.28, 95% CI 1.83–5.88). In addition, the association showed high heterogeneity (*I* ^2^ = 79.9%). The forest plots of the frequency of thyroid disease in patients with sarcoidosis compared with the controls are presented in Fig. 2. The prevalence of clinical thyroid disease was higher in patients with sarcoidosis than in the controls (OR 2.66, 95% CI 1.44–4.90) (Fig. 3). Furthermore, we investigated the relationship between different thyroid antibodies and sarcoidosis. The prevalence of TPOAb positive was higher in sarcoidosis patients than in the controls (Table 3). As a result of the studies involving different races, subgroups were analyzed based on the different races on which the studies were performed. The subgroup analysis results showed a higher correlation in Caucasian than Asian (Asian: OR 3.05, 95% CI 0.84–11.10; Caucasian: OR 3.74, 95% CI 1.51–9.24) (Fig. 4).

**Table 3:** Results of the subgroup analysis of the correlation between different thyroid antibodies and sarcoidosis.

	Study (n)	Odds ratio	95% Confidence intervals	Method	Heterogeneity	
					*I*^2^	*P*
TgAb positive	Chen & Moller (4)	2.17	0.56–8.32	Random, Mantel-Haenszel	80.10%	0.001
TPOAb positive	Chen & Moller (4)	2.95	1.53–5.68	Random, Mantel-Haenszel	50.3%	0.134

TgAb, thyroglobulin antibody; TPOAb, thyroid peroxidase antibody.

### Sensitivity analysis and publication bias

**Figure 5:**
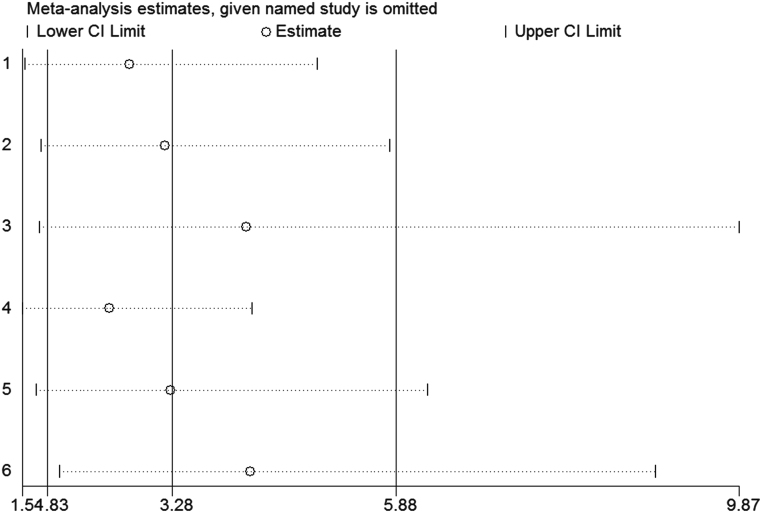
The sensitivity analysis results.

Using the sensitivity analysis by excluding individual studies one by one, the results showed little difference, suggesting that the results of this study were relatively credible (Fig. 5). A comprehensive search of the articles obtained from the database was performed. Begg’s test was also performed, and the results showed that the possibility of publication bias was small.

## Discussion

This meta-analysis is the first to evaluate the pooled incidence of thyroid disease in sarcoidosis patients. Some studies have assessed the risk of thyroid disease in sarcoidosis patients; however, due to inconsistent results in these studies, it is difficult to determine the relationship between sarcoidosis and thyroid disease. In this meta-analysis, 6 independent studies were included and analyzed. The results showed that the incidence of thyroid disease was significantly increased in sarcoidosis patients than in the controls (OR 3.28, 95% CI 1.83–5.88).

The prevalence and incidence rate of sarcoidosis is related to age, gender, race, and region. It is reported that Afro-Caribbeans and African Americans are the two ethnic groups most affected by sarcoidosis (21). According to data from Sweden, the total prevalence rate is 160 per 100,000 people (22). The prevalence of sarcoidosis in African Americans is 141 per 100,000 people, while Caucasians in the same region are only 49.8 per 100,000 people (23). Whether sarcoidosis can be classified as an autoimmune disease has always been controversial. Some researchers believe that the gender orientation of sarcoidosis is not strong, specific autoantibodies have not been found, and it is most common in the lungs and thoracic lymph nodes rather than common autoimmune diseases such as skin and joints involving organs. Therefore, it cannot be classified as an autoimmune disease (24). But there is also a lot of evidence that suggests similarities between sarcoidosis and autoimmune diseases, such as lupus erythematosus, rheumatoid arthritis, Sjogren’s syndrome, and other autoimmune diseases (25). HLA genotypes are also related to disease susceptibility and prognosis. In a national-level questionnaire-based study conducted on 3835 self-reported sarcoidosis patients, they educated that hypothyroidism is a prevalent coexisting disease in sarcoidosis patients and sarcoidosis patients with hypothyroidism have more multiple organ presentations, such as skin, joints, eyes, liver, and lacrimal glands showing sarcoid presentations than typical pulmonary sarcoidosis (26).

Both sarcoidosis and thyroid disease have been associated with HLA genes. Brewerton first reported an HLA-88 association with acute sarcoidosis (27). Another study showed HLA-B and HLA-DRB1 association with both sarcoidosis and thyroid disease (28, 29, 30). Papi demonstrated that class I HLA and class II might play complementary roles in promoting both thyroid disease and sarcoidosis through a common immunopathogenic pathway (31). The association between Hashimoto’s thyroiditis and sarcoidosis could be due to increased thyroid-specific T-cell activation caused by increased expression of Th1/Th17 cells in both diseases (32).

This meta-analysis aimed to statistically evaluate the incidence of thyroid disease in sarcoidosis patients. However, this work has some limitations. Due to the lack of randomized controlled trials, the studies in this meta-analysis were only case-control studies. Some studies used volunteers with other skin diseases, except for autoimmune disorders, as controls instead of healthy volunteers. In addition, the variability of the test methods used in each study, with time points varying by more than 20 years, may have an impact on the results. Therefore, the results of this meta-analysis should be interpreted cautiously. More high-quality studies are needed to better support the association between thyroid disease and sarcoidosis.

## Conclusion

This systematic review is the first to evaluate the pooled incidence of thyroid disease in sarcoidosis patients. The results of our meta-analysis support the hypothesis that the prevalence of thyroid disease is higher in patients with sarcoidosis compared to controls, which suggests that sarcoidosis patients should be screened for thyroid disease.

## Supplementary Materials

Table S1

Table S2 ROBINS-I Assessment of Study Bias for Included Studies

## Declaration of interest

The authors have nothing to disclose.

## Funding

This research was financially supported by the Natural Science Foundation of Jiangsu Province http://dx.doi.org/10.13039/501100004608 (grant No. BK2 http://dx.doi.org/10.13039/5011000102380200204).

## Ethical approval and consent to participate

Not applicable.

## Availability of data and materials

All data relevant to the study are included in the article or uploaded as supplemental information.

## Consent for publication

Not applicable.

## Ethical guidelines

Not applicable.

## Author contribution statement

Caihong Xin: Conceptualization, Methodology; Jing Xie: Data curation, Writing-Original draft preparation; Lijuan Niu: Software, Validation. Huaying Fan: Visualization, Investigation. Xin Sun: Writing-Reviewing and Editing.
