# Supplementary material for: Increased incidence of thyroid disease in patients with sarcoidosis: a systematic review and meta-analysis
Source: Endocr Connect. 2023 Aug 1;12(9):e230157. doi: 10.1530/EC-23-0157 (PMC10448568; doi:10.1530/EC-23-0157)
Supplement: Table S2 ROBINS-I Assessment of Study Bias for Included Studies [file supplementary_table_2.pdf]

Table S2 ROBINS-I Assessment of Study Bias for Included Studies

	Confounding	Selection of participants into the study	Classification of exposures	Deviations from intended exposures	Missing data	Measurement of outcomes	Selection of the reported result	Risk of bias scores
Hirotoshi Nakamura, 1997	Moderate	Low	Low	Low	Low	Low	Moderate	Moderate
Ioannis Llias, 1998	Moderate	Low	Moderate	Low	Low	Low	Moderate	Moderate
Alessandro Antonelli, 2005	Low	Low	Low	Low	Low	Low	Low	Low
Foteini Malli, 2012	Low	Low	Low	Low	Low	Low	Low	Low
Adam Nowinski, 2015	Low	Low	Low	Low	Low	Low	Low	Low
Chihung Wu, 2016	Moderate	Moderate	Low	Low	Low	Low	Low	Moderate
